# U.S. young adults’ family-building intentions in the event of infertility and knowledge of associated costs

**DOI:** 10.3389/fgwh.2025.1538455

**Published:** 2025-06-27

**Authors:** Jean Marie Place, John Horowitz, Minh Nguyen, Maya Guinn, Brennan Peterson

**Affiliations:** ^1^ Department of Nutrition and Health Science, Ball State University, Muncie, IN, United States; ^2^ Department of Economics, Ball State University, Muncie, IN, United States; ^3^ Ball State University, Muncie, IN, United States; ^4^ Department of Marriage and Family Therapy, Chapman University, Orange, CA, United States

**Keywords:** infertility, cost, IVF, adoption, fertility awareness, intentions

## Abstract

**Introduction:**

Infertility affects approximately 8.5% of married women in the United States, yet there is limited understanding of how young adults anticipate and prepare for potential barriers to biological parenthood. As reproductive planning increasingly intersects with social, medical, and financial considerations, it is important to examine how emerging adults perceive and approach family-building options. This study aims to explore college students' intentions regarding childbearing, their openness to non-traditional family-building methods, and their awareness of the associated financial costs.

**Methods:**

A cross-sectional survey was administered to undergraduate students (mean age = 20) at a Midwestern public university. Most participants had not previously attempted to conceive. The survey assessed fertility awareness, preferences for family-building in the context of infertility, and cost estimations for various non-traditional options, including adoption, in vitro fertilization (IVF), and surrogacy.

**Results:**

The majority of participants (86%) expressed a desire for biological children, with an average intended family size of 2.3 children. Among non-traditional options, private domestic adoption was the most preferred (58.3%), followed by public adoption (53.0%) and IVF (42.2%). Surrogacy using donor gametes was the least preferred (9.7%). Cost awareness was generally low: only 16.7% of estimates for domestic adoption and 48% for international adoption fell within 75%–125% of actual cost ranges. Gender differences emerged, with women showing greater openness to alternative family-building methods and more accurate cost perceptions, while men reported higher confidence in their reproductive planning.

**Discussion:**

These findings suggest that while college students are generally interested in parenthood, they may lack adequate knowledge about the financial and logistical realities of non-traditional family-building options. The gender differences observed highlight the need for targeted educational interventions. Enhancing fertility literacy and financial preparedness among young adults could support more informed and realistic family planning decisions.

## Introduction

Childbearing intentions arise from a complex interplay of individual and contextual factors operating at both micro and macro levels (1). In the United States, 85%–87% of reproductive-aged men and women expect to have at least one biological child—that is, a child genetically related to them [Centers for Disease Control (CDC), National Survey of Family Growth (NSFG), 2017–2019, B Listing]. However, many individuals and couples face barriers to achieving this goal due to infertility, which the World Health Organization (2) defines as the inability to conceive after 12 months or more of regular, unprotected intercourse. Globally, infertility affects 17.5% of individuals over their reproductive lifespan, while in the U.S., 9.7 million women aged 15–49 (13.4%) experience difficulty getting pregnant or carrying a pregnancy to term (CDC, NSFG, 2017–2019, I Listing). From 1982 to 2010, impaired fecundity, known as having difficulty carrying pregnancies to live term, increased among all women in the United States but has since remained relatively stable (3, 4).

The diagnosis of infertility is associated with increased psychological distress, including depressive symptoms, feelings of stigma, and the search for meaning in life in both men and women (5). In the United States, women who had less income, were older, had less education, were non-Hispanic Black, or had not received sexual and reproductive health services were more likely to experience infertility (4). The burden of infertility has been increasing over time for men (6), and advanced paternal age, which has been steadily rising in the U.S., particularly among college-educated men, is associated with difficulties conceiving, increased miscarriage rate, and chromosomal issues in offspring (7, 8).

Socio-demographic shifts play a role in these childbearing challenges. When men and women marry at later ages, pursue higher education, and achieve career goals, they may delay attempts to conceive until the ages when their fertility declines (9, 10). Additionally, many environmental and lifestyle factors contribute to impaired fecundity, such as substance abuse, personal health, well-being imbalance (i.e., nutrition, stress, exercise), radiation exposure, and pollution (11). Many young adults may not fully understand the impact of age or other environmental and lifestyle choices on fertility. In studies across the globe (12, 13), university students consistently demonstrate a lack of knowledge of human reproduction and fecundity. Such misinformation can threaten young adults’ family-building goals and lead to involuntary childlessness due to overestimating women's reproductive window and underestimating the harmful effects of certain lifestyle choices.

While the primary form of childbearing for most couples is spontaneous, non-assistive conception, “non-traditional family-building options” are sometimes pursued after a couple or individual fails to conceive spontaneously due to infertility. For the purposes of this study, non-traditional family building is defined as behavioral attempts to have a child if spontaneous, natural conception does not occur. This can include the use of assisted reproductive treatments (ART) using one's own gametes, using donated third-party gametes from one or both partners, adoption, and surrogacy.

The use of ART has been increasing in the United States. In 2023, 157,120 treatment cycles involving egg retrieval and embryo transfer were initiated with patients' own eggs (14). Although gamete donation is increasing worldwide (15), nearly all men and women seeking fertility treatments prefer genetic parenthood to non-genetic parenthood (16). Consequently, it is estimated that third-party reproduction is used in only 16.1% of ART cylces in the United States (15). Building a family through adoption is an additional option, with 19,658 private adoptions taking place in the United States in 2020, 1,618 international adoptions, and 57,802 public adoptions through foster care (17).

Over the past two decades, a growing body of literature has examined fertility awareness, family planning and family-building options, and how individual and environmental risk factors influence fertility (18). While these studies have documented the public's lack of knowledge of human reproduction and fecundity, less research has examined the public's awareness of non-traditional family-building options. Being faced with having to use non-traditional family-building options can cause substantial psychological distress by diverging from an original plan of non-assistive conception and/or biological parenthood (19). In a context where many non-traditional family-building options have high costs that insurance does not cover—for example, an estimated 85% of *in vitro* fertilization (IVF) costs are out-of-pocket (20), and the per-person cost estimate for a successful outcome with IVF is $61,377 (21)—young people's understanding of the financial implications of these options should be considered. Studies have shown young adults facing infertility are surprised and unprepared for the costs related to non-traditional family building and have substantial anxiety about covering costs (e.g., depleting savings, incurring debt) (22). It is important to consider young adults' knowledge of and intentions toward non-traditional family-building options to provide them with helpful information in their family planning decision-making. Considering how knowledge of and intentions toward non-traditional family-building options may differ by sex (male/female) can help provide targeted financial planning communication to those needing the information.

## Methods

### Participants and procedures

We conducted a cross-sectional study among undergraduate college students at a midwestern public university. Data collection was two-pronged and occurred over three years (2018–2020). Only participants aged 18 and over were allowed to participate. First, we gained permission from two instructors who teach a wide selection of students from various majors in core university classes to come into the classes, discuss the study, and invite students to read the informed consent and complete the 15-minute survey during the class period. To avoid coercion, we informed the students that participation was completely voluntary and that they could withdraw without penalty or impacting their grades. Instructors were not aware of which students participated. The second approach recruited participants from an undergraduate subject pool, where students provided consent to participate and completed at least one online survey for which they met eligibility criteria. Our online survey with informed consent was available for students to complete during three two-week periods. The study received ethics approval from Ball State University institutional review board (IRB # 1176744-2).

In total, 1,048 students completed the survey. We did not collect data on students who received the invitation but chose not to reply. Since the analysis focuses on male and female differences in family-building intentions, we only included participants who chose male or female in the survey. This left a total of 962 participants in the analysis.

### Instruments

The current study used selected questions from the Swedish Fertility Awareness Questionnaire, which has been shown to have face validity and reliability (23). The measure was selected due to its wide use in international studies of fertility awareness and parenting intentions in the United States (24), Hong Kong (25), Denmark (26), and Mexico (13). It has also been used to assess the fertility awareness of obstetric and gynecology (OB-GYN) medical residents in the United States (27). Our 16-item survey combined 13 questions from the Swedish Fertility Awareness Questionnaire with three additional questions designed by the researchers. The original instrument assessed participants attitudes towards having children including the the desired number of children, their preferred ages when having children, and the confidence they would have that number of children at at their desired ages. The instrument also assessed general fertility issues such as age at which female fertility declines and the chance of having a child after using IVF. The additional questions were assessed for face and content validity by an expert in health economics and pilot-tested with a sample of undergraduate college students at another university. The feedback provided led to minor revisions in item wording and response options. The first additional question used a 5-point Likert scale to assess how much non-traditional family building options cost, with responses ranging from most to least expensive. The second additional question assessed knowledge of adoption costs for adoptions completed in the United States vs. internationally, using an open-response format. The third additional question assessed behavioral intentions in the event of infertility and asked participants to think hypothetically. For each option [e.g., undergoing *in vitro* fertilization, working with a surrogate to carry a pregnancy using one's own gametes, adopting from the public sector (i.e., foster care) that deals with adoptions in the United States, etc.], participants could select a response option using a 5-point scale from extremely unlikely to extremely likely.

### Statistical analyses

We calculated descriptive statistics by gender for participants' demographic characteristics, knowledge and awareness of family-building costs, and intentions regarding both traditional and non-traditional family-building options. We also report the distribution of students' estimated costs for domestic and international adoptions. To assess whether gender differences in these outcomes are statistically significant, we conducted *t*-test analyses. All analyses were performed using STATA 16.

## Results

Table 1 presents the demographic characteristics of the sample population. Approximately 50% of participants identified as female and 50% as male. Ninety-three percent of males and 90% of females identified as heterosexual. About 80% of participants were White, 7% Black, 3% Asian, 2% Latino, and 6% other. Eighty-two percent of male students and 77% of female students reported White race. On average, sample participants were 21 years old, and their median age was 20. Approximately 51% of the sample was single, 47% were married or in a committed relationship, and 2% reported another status, such as divorced or other. About 67% of students were business majors, partly because sample participants were recruited from marketing classes where one of the assignments was to participate in a study of their choice. The rest of the sample was evenly distributed from majors in other colleges.

**Table 1:** Demographic characteristics.

Characteristic	All	Male	Female
Gender identity (%)			
Female	50.42 [47.25, 53.58]		
Sexual orientation (%)			
Heterosexual	92.74 [91.07, 94.40]	93.07 [90.96, 95.180]	90.31 [90.51, 95.63]
Race/Ethnicity (%)			
White	80.46 [77.95, 82.97]	82.39 [78.96, 85.82]	78.56 [74.89, 82.22]
Black	7.38 [5.73, 9.04]	7.55 [5.17, 9.93]	7.22 [4.91, 9.53]
Asian	3.01 [1.93, 4.1]	2.1 [0.81, 3.39]	3.91 [1.55, 4.64]
Latino	2.39 [1.42, 3.36]	1.68 [0.52, 2.83]	3.09 [1.55, 4.64]
Age			
Average age	20.81 [20.59, 21.02]	20.81 [20.50, 21.12]	20.81 [20.51, 21.11]
Relationship status (%)			
Single	51.04 [47.87, 54.20]	53.88 [49.39, 58.37]	48.25 [43.78, 52.71]
Married	2.81 [1.76, 3.85]	2.31 [0.95, 3.66]	3.30 [1.70, 4.89]
In a committed relationship or engaged	44.28 [41.14, 47.73]	42.35 [37.90, 46.80]	46.29 [41.73, 50.64]
Other	1.87 [1.01, 2.73]	1.47 [0.38, 2.55]	2.27 [0.94, 3.60]
Major (%)			
Health-related	7.90 [6.19, 9.61]	10.27 [5.54, 13.01]	5.57 [3.52, 7.61]
Business-related	66.74 [63.75, 69.72]	69.18 [65.02, 73.34]	64.33 [60.05, 68.61]
Natural Science	6.34 [4.80, 7.88]	9.64 [6.99, 12.30]	3.09 [1.55, 4.64]
Art-related	6.44 [4.89, 8.00]	2.73 [1.26, 4.19]	10.10 [7.41, 12.79]
Communications	7.80 [6.10, 9.49]	6.71 [4.46, 8.96]	8.87 [6.33, 11.40]
Others, mainly social science	4.78 [3.43, 6.13]	1.47 [0.38, 2.55]	8.04 [5.61, 10.47]

95% confidence intervals are shown in brackets.

### Intentions about traditional family building

Table 2 shows the results for participants' intentions about traditional family building. Nearly 86% of participants intended to have children, and of those who desired to have children, the average number of children desired was 2.3. For those already with children, the age at which they had their first child was about 23. For those who planned to have children, the planned date to have their first child was approximately 27. Only 24.8% of the respondents were confident in their plans for the number and timing of children. However, males were more confident in their plans for the number and timing of children (30.4%) than females (19.4%).

**Table 2:** Intentions about traditional family building.

Level of importance	All	Male	Female	*p*-value
Whether plan to have children (%)	85.9	87.8	83.9	0.080
	[83.7, 88.1]	[84.9, 90.8]	[80.6, 87.2]	
Number of children desired^a^	2.3	2.2	2.4	0.125
	[2.2, 2.4]	[2.2, 2.4]	[2.2, 2.5]	
Age at first-child^b^	23.2	24.2	22.6	0.319
	[21.6, 24.8]	[20.8, 27.6]	[20.7, 24.5]	
Age for first-child (Planned)	27	27.4	26.6	0.000
	[26.8, 27.2]	[27.2, 27.7]	[26.4, 26.8]	
Age at last-child	32.6	32.82	32.28	0.028
	[32.3, 32.8]	[32.4, 32.8]	32.0, 32.6]	
Confidence in achieving the number of children at desired ages^c^ (%)	24.8	30.4	19.38	0.000
	[22.1, 27.6]	[26.3, 34.5]	[15.9, 22.9]	

^a^ Among participants who plan to have children.

^b^ Among participants who have children already (*N* = 26).

^c^ Confidence includes responses “confident” and “very confident” that they can achieve the number of children at the desired ages.

95% confidence intervals are shown in brackets. *p*-values below 0.05 show statistically significant values, indicating a statistically significant difference.

### Intentions about non-traditional family building

Table 3 shows the participants' intentions regarding non-traditional family building in the event of infertility. Participants were asked how likely they would be to choose a specific option of non-traditional family building from 1 (extremely unlikely) to 5 (extremely likely). They are considered to have intentions to choose an option if they selected 4 or 5. Private domestic adoption was the most commonly reported method of non-traditional family building (58.3%), followed by adoption from foster care (53.0%), *in vitro* fertilization (IVF) (42.2%), international adoption (39.9%), surrogacy with own genetic material (28.5%), and choosing not to have children (23.5%). Participants were least likely to select surrogacy with donor gametes (9.7%). Both males and females had the same order of intentions in the event of infertility. However, significant gender differences were present in five of the seven non-traditional family-building options in terms of their level of endorsement. Females are much more likely than males to report that they intend to pursue non-traditional family-building methods including domestic adoptions (68.5% for females vs. 48.0% for males), adoption from foster care (58.8% for females vs. 47.2% for males), IVF (51.1% for females vs. 33.1% for males), international adoptions (50.9% for females vs. 28.7% for males), and surrogacy with their own gametes (egg or sperm) (33.3% for females vs. 23.7% for males). These differences are statistically significant at the.05 level.

**Table 3:** Intentions about non-traditional family building.

In event of infertility, intention to pursue:	All	Male	Female	*p*-value
Private-domestic adoption	58.3	48	68.5	0.000
	[55.2, 61.4]	[43.5, 52.5]	[64.3, 72.6]	
Adoption from foster care	53	47.2	58.8	0.000
	[49.9, 56.2]	[42.7, 51.7]	[54.4, 63.2]	
IVF	42.2	33.1	51.1	0.000
	[39.1, 45.3]	[28.9, 37.4]	[46.7, 55.6]	
International adoption	39.9	28.7	50.9	0.000
	[36.8, 43.0]	[24.6, 32.8]	[46.5, 55.4]	
Surrogacy with own gametes (egg or sperm)	28.5	23.7	33.2	0.001
	[25.6, 31.3]	[19.9, 27.5]	[29.0, 37.4]	
Choose not to have children	23.5	23.5	23.5	0.993
	[20.8, 26.2]	[19.7, 27.3]	[19.7, 27.3]	
Surrogacy with donated gametes (egg or sperm)	9.7	9.9	9.5	0.847
	[7.8, 11.5]	[7.2, 12.5]	[6.9, 12.1]	

95% confidence intervals are shown in brackets. *p*-values below 0.05 indicate a statistically significant differences between male and female respondents.

### Perception of non-traditional family building costs

Table 4 shows the participants' ranking of the cost of various non-traditional family-building options. Approximately 50% of males and 45% of females thought that one cycle of *in vitro* fertilization was the most expensive option (IVF), followed by international infant adoption (21% male; 23% female), contracting the services of a female surrogate to carry a pregnancy (16% male; 20.6% female), and infant adoption in the United States (8.6% male; 6.7% female). Lastly, roughly 5% of males and females thought that infant adoption through foster care was the most expensive.

**Table 4:** Participants’ cost ranking of various non-traditional family-building options^a^.

Non-traditional family-building option	% ranking option most expensive			% ranking option 2nd most expensive			% ranking option 3rd most expensive			% ranking option 4rd most expensive			% ranking option least expensive		
	Male	Female	*p*-value	Male	Female	*p*-value	Male	Female	*p*-value	Male	Female	*p*-value	Male	Female	*p*-value
One cycle of *in vitro* fertilization (IVF)	49.8	45.0	0.458	20.0	26.9	0.007	8.8	10.5	0.289	11.0	11.4	0.654	10.5	6.3	0.042
Infant adoption international	20.7	23.0	0.243	19.5	18.8	0.958	36.4	35.1	0.922	9.5	12.8	0.083	13.8	10.3	0.182
Contracting the services of a female surrogate	16.0	20.6	0.040	36.0	33.3	0.755	14.1	18.8	0.031	15.5	12.3	0.284	18.6	15.0	0.272
Infant adoption within the US	8.6	6.7	0.404	15.7	16.6	0.533	22.6	24.6	0.296	41.9	44.7	0.168	11.2	7.4	0.087
Infant adoption through foster care	5.0	4.7	0.956	8.8	4.5	0.017	18.1	11.0	0.007	22.1	18.8	0.384	46.0	61.1	0.000

^a^ The order of most expensive to least expensive non-traditional family building options, according to published data, is (1) Surrogacy with an average cost of $125,000 to $175,000 (28); (2) International infant adoption with an average cost between $20,000 and $50,000 (29, 30); (3) Domestic infant adoption with an average cost between $20,000 and $45,000 (29, 30); (4) IVF with an average cost of $12,400 to $25,000 (21, 31, 32); (4) Infant adoption through foster care with an average of $0 to $2,600. *p*-values below 0.05 indicate a statistically significant differences between male and female respondents.

Conversely, when identifying the least expensive option, 46% of males and 61.1% of females thought infant adoption through foster care was the least expensive option. This was followed by surrogacy (18.6% of males; 15% of females), international adoption (13.8% of males; 10.3% of females), and domestic adoption in the U.S. (11.2% of males; 7.4% of females). Lastly, 10.5% of males and 6.3% thought that IVF was the least expensive option.

Table 5 shows the quintiles of participants' domestic and international adoption cost estimates by gender. We excluded outliers with cost estimates of more than $300,000 (8 observations) or less than $10 (6 observations).

**Table 5:** Estimates of domestic and international adoption costs^a^.

Knowledge about costs	Domestic adoption ($)				International adoption ($)			
	Overall	Male	Female	*p*-value	Overall	Male	Female	*p*-value
Quintile 1	1,552	1,510	1,632	0.469	2,441	2,397	2,528	0.602
	[1,396, 1,708]	[1,317, 1,703]	[1,360, 1,903]		[2,207, 2,675]	[2,102, 2,691]	[2,139, 2,918]	
Quintile 2	7,843	7,804	7,892	0.752	9,156	9,204	9,086	0.574
	[7,573, 8,113]	[7,437, 8,172]	[7,489, 8,295]		[8,952, 9,360]	[8,941, 9,468]	[8,758, 9,414]	
Quintile 3	17,981	18,088	17,899	0.663	19,032	19,118	18,959	0.781
	[17,557, 18,405]	[17,457, 18,719]	[17,318, 18,480]		[18,474, 19,590]	[18,260, 19,977]	[18,217, 19,702]	
Quintile 4	28,333	27,660	28,828	0.0096	35,356	34,985	35,577	0.512
	[27,888, 28,779]	[26,919, 28,400]	[28,295, 29,361]		[34,498, 36,214]	[33,625, 36,346]	[34,459, 36,695]	
Quintile 5	63,994	70,407	61,108	0.236	81,440	85,397	79,273	0.398
	[56,840, 71,148]	[54,561, 86,253]	[53,450, 68,767]		[74,580, 88,301]	[72,142, 98,616]	[71,385, 87,089]	

^a^ For reference, domestic infant adoption costs on average between $20,000 and $45,000 (29, 30), and international infant adoption costs between $20,000 and $50,000 (29, 30). 95% confidence intervals are shown in brackets. *p*-values below 0.05 indicate a statistically significant differences between male and female respondents.

Our results indicate that only 16.7% of student estimates were within the 75%–125% range of the actual costs for domestic adoptions, and 48% were within the 75%–125% range of the actual costs for international adoptions.^1^ Among participants who provided domestic adoption cost estimates, those in the lowest 20% (Quintile 1) reported a median estimated cost of $1,552. Participants in the middle 20% (Quintile 3) reported a significantly higher median of $17,981, while those in the highest 20% (Quintile 5) estimated a median cost of $63,994. For international adoption, participants in the lowest 20% of cost estimates (Quintile 1) reported a median estimated cost of $2,441. In the middle 20% (Quintile 3), the median estimate was $19,032, and in the highest 20% (Quintile 5), the median rose substantially to $81,440.

## Discussion

Young people may be unprepared for the possibility of infertility, with prior research suggesting that people lack fertility awareness (24, 33), particularly as it relates to modifiable and nonmodifiable risk factors for infertility, consequences of delayed childbearing, and fertility health (12, 34). While infertility prevention efforts should continue, providing accurate information to help young people prepare in case of a need for non-traditional family-building options is an essential part of sexual and reproductive education. Delbaere et al. (35) indicate that even with fertility awareness education programs, people may need additional education about fertility interventions' costs and success rates and the information associated with non-biological family-building options (36). In the event of infertility, patients may face complex, risk/benefit decisions regarding debt, loans, and where and how to spend resources and assets (37). Studies indicate that low health cost literacy is associated with patients being unprepared for bills, making uninformed treatment decisions, and struggling to access financial support resources (38).

In the event of infertility, our participants ranked private domestic adoption as the most likely family-building option, followed by adoption from the public sector (i.e., foster care), IVF, and international adoption. Because infertility is ranked as the primary motive for pursuing private domestic adoption (30), it is essential to communicate possible financial and systemic challenges with this family-building option and strategize ways to address obstacles. Financial deterrents commonly include high adoption fees and additional expenses, such as medical, court, legal, and attorney expenses (39). According to the Adoption Cost and Timing Survey, between 2016 and 2017, the average private domestic adoption cost through an agency was $43,239, and the average international adoption cost for adoptive parents in the U.S. was $44,000 (40). In our study, 60% of students (those in Quintiles 1–3) estimated that the cost of either domestic or international adoption was less than $20,000, which is notably less than actual, average costs (29, 30). Additionally, 80% of students (Quintiles 1–4) believed that domestic adoption costs were under $30,000 and international adoption costs were under $40,000. Based on participants' domestic and international adoption cost estimates (Table 5), most participants thought adoption costs were lower than they were. In our survey, less than a quarter of participants perceived the costs of international adoption—as they understood them—as very expensive, and less than 10% perceived domestic adoption as very expensive (Table 4). This stands in contrast to a national sample where over half of adoptive parent respondents stated that prior to their adoption, they perceived cost to be a barrier (30). While 46% of males and 61% of females of respondents correctly perceived infant adoption through foster care as the least costly option, it may not be a “no cost” option. Pereption of the cost of foster care differs significantly between males and females, with a greater percentage of females more likely to rank it as the least expensive option compared to males. Data from the Adoption Cost and Timing Survey indicate that the average costs reported by families who adopted from foster care were $2,938 due to home study fees, document preparation and other paperwork fees, and other expenses. These findings underscore the role of awareness of and pre-planning for adoption as a non-traditional family building option, including providing knowledge on available tax credits, resimbursements, subsidies, employer benefits, and adoption loans and grant programs. In addition to costs, limitations to private and independent adoptions may also include uncertainty from both parties (i.e., birth parents and prospective parents) and state restrictions for public adoptions (39), including strict application conditions and interminable waiting periods (41). Additional barriers include a shortage of obtainable information for families looking to adopt, poor post-adoption support, and inadequate recruiting programs to locate and support birth parents (42). As most prospective parents are aged 40 or older, age discrimination is a significant barrier these couples face (41). These barriers have prevented some prospective couples from continuing through with the adoption process (42).

Other avenues for family building include seeking fertility treatments such as *in vitro* fertilization (IVF) using one's own or donated gametes. Assisted reproductive technologies (ART) are procedures that manipulate the eggs and/or sperm or embryos to aid in conception of a pregnancy, specifically for those facing infertility. Our results suggest IVF is one of the top three options considered by respondents in the event of infertility. While ART is successful for some, many young couples overestimate the effectiveness of IVF and underestimate the challenges that are involved (43). High out-of-pocket costs are a barrier for people seeking to build their family through IVF, with cost identified as a leading reason why women say they could not obtain the fertility care they needed (44). Cost is a particular barrier for low-income women compared to high-income women (44). According to the National Council of State Legislatures, the average IVF cycle can cost between $12,000 to $17,000. Additional medication costs make the process close to $25,000. The costs are exacerbated given realities of fertility care in the U.S.: most couples will need more than one round of IVF, private insurance plans can restrict access to fertility care, and there is limited grant assistance (45). In our survey, just about half (49.8% of males; 45% of females) of participants thought one cycle of IVF was the most expensive option and 10.5% of males and 6.3% of females perceived IVF as the least expensive option, with the later being a statistically significant difference between males and females. Research suggests that among couples who have successfully conceived via IVF, the treatment is considered expensive but worth it (46). People seeking the use of IVF may also encounter challenges in accessing services, psychological distress following unsuccessful treatment, and maternal and infant risks for multiple births (43). These obstacles can lead to disappointment and admission of defeat for couples looking to build a family. The Amerian Society for Reproductive Medicine (ASRM) encourages public education about the prevention, signs, and treatments of infertility, particularly for underserved areas and racial and ethnic minorities (47).

In our study, women were far more likely than men to consider a range of non-traditional family-building options. Men, in particular, had very little knowledge of the actual costs of non-traditional family-building options. In some cases, men and women significantly differed in their perceived expense of family-building options. A lack of knowledge or “unmet information needs” on non-traditional family building is associated with higher decisional conflict in making fertility-related health care decisions (48). According to systematic reviews, most men sampled have inadequate knowledge about human reproduction, lack understanding about the limits and influences of fertility, and overestimate the chance of spontaneous and assisted conception (12, 49). Although men report a widespread desire for fatherhood, there is a widening gap between the desired timeframe for parenthood and the ideal reproductive window (24). Thus, increasing the acceptance and understanding of non-traditional family-building options should be a focus among this population.

Finally, educational efforts in schools and the health care systems regarding fertility and the availability of alternative family building options are needed. The aim of such efforts is to increase the accuracy of fertility knowledge among men and women in society that can correspond with increased flexibility in childbearing intentions so they align with childbearing decisions. It is encouraging that past research finds the majority of OB-GYNs believe having fertility-informed discussions with patients is vital to their care, and we support the recommendations of Yu and colleagues that these discussions be part of an annual well-woman exam (27). Community-level public programs and education that increase social awareness of the physical, emotional, and financial burdens of infertility can improve men and women's ability to make informed reproductive decisions based on accurate knowledge of fertility and alternative family-building options.

### Limitations

Several factors limit the present study. First, the findings may reflect the self-selection bias inherent in convenience sampling. Students who are more interested in fertility issues may be more likely to respond to the study invitation. Second, the study may contain non-response bias because we did not collect data on students who chose not to participate and thus could not compare the characteristics of participants and non-participants. Third, despite being a relevant non-traditional family-building option, we failed to include a response for donor insemination or oocyte donation. Likewise, we did not differentiate between IVF using a couple's own gametes and IVF as a type of third-party reproduction where donor gametes are used. Fourth, respondents were mainly business majors from a single midwestern university, which means respondents came from a limited geographical area and could have thus limited socioeconomic backgrounds, cultures, and ages. Most had no previous attempts at conceiving. On the other hand, 2.7% of survey participants already have children. However, their opinions regarding infertility may also not accurately reflect reality, as they may not have considered how their fertility changes as they age, alternative family-building options, or the cost of those options.

These omissions limit the study's generalizability. In interpreting the findings, it is important to consider that the results may not be reflective of a wider U.S. population. Nevertheless, the options included in the survey provide preliminary findings, although the picture is incomplete and limited in scope. Future research should include additional non-traditional family-building options and a broader demographic sample. Furthermore, looking at how family-building intention and knowledge levels change over time and by marital status or based on attempts at conception would be useful.

## Conclusion

Educational efforts should be focused on helping young people interested in building families become aware of the limitations of biological childbearing, understand the financial costs of non-traditional family-building options, and prepare for these costs when using non-traditional family-building options in the event of involuntary childlessness (50, 51).

## Data Availability

The raw data supporting the conclusions of this article will be made available by the authors, without undue reservation.

## Footnotes

^1^The 75-125% range for domestic adoption cost is ($29,250, $48,750) and the analogous range for international adoption cost is ($15,000, $62,500)
